# Structural brain alterations and predictors of clinical improvement in functional cognitive disorder after concussion^☆^

**DOI:** 10.1016/j.nicl.2025.103936

**Published:** 2025-12-19

**Authors:** Christiana Westlin, Mathilde Rioux, Jaqueline Lee, William Panenka, Daniela J. Palombo, Rebecca M. Todd, Noah D. Silverberg, David L. Perez

**Affiliations:** a Functional Neurological Disorder Research Group, Department of Neurology, Massachusetts General Hospital, Mass General Brigham Integrated Healthcare System, Harvard Medical School, Boston, MA 02114, USA; b Department of Psychiatry, Massachusetts General Hospital, Mass General Brigham Integrated Healthcare System, Harvard Medical School, Boston, MA 02114, USA; c Athinoula A. Martinos Center for Biomedical Imaging, Massachusetts General Hospital, Harvard Medical School, Boston, MA 02129, USA; d Department of Psychology, The University of British Columbia, Vancouver, British Columbia, Canada; e British Columbia Neuropsychiatry Program, The University of British Columbia, Vancouver, British Columbia, Canada; f Department of Psychiatry, The University of British Columbia, Vancouver, British Columbia, Canada; g Djavad Mowafaghian Centre for Brain Health, The University of British Columbia, Vancouver, British Columbia, Canada; h Rehabilitation Research Program, Centre for Aging SMART, Vancouver Coastal Health Research Institute, Vancouver, British Columbia, Canada

**Keywords:** Functional cognitive disorder, Functional neurological disorder, Structural MRI, Concussion, Amygdala

## Abstract

Functional cognitive disorder (FCD) is an under-studied yet likely prevalent subtype of functional neurological disorder (FND), with neural mechanisms that remain poorly understood. While studies of other FND subtypes have revealed structural and functional brain alterations across several large-scale brain networks, no study to date has investigated neural alterations in FCD. Here, we examined structural grey matter brain differences as measured from magnetic resonance imaging in 37 adults with FCD after concussion compared to 25 post-concussion controls. We compared *FreeSurfer*-based cortical thickness and subcortical volumes between groups and investigated associations between structural measures and memory symptoms. A subset of participants with FCD (n = 24) participated in a randomized controlled trial of cognitive-behavioral therapy vs. cognitive rehabilitation, and relationships between baseline structural measures and treatment outcomes were evaluated. All primary analyses were adjusted for age, sex, estimated IQ, and loss of consciousness, and corrected for multiple comparisons. No significant between-group differences in cortical thickness or subcortical volume were observed. However, across all participants, greater functional memory symptom severity was associated with larger right amygdala volume, which remained significant adjusting for depression scores, anxiety scores, and psychotropic medication use. *Post-hoc* analyses identified that this observation was driven by differences in lateral, basal, and paralaminar amygdalar nuclei. Additionally, greater improvement in memory symptoms following treatment was associated with increased pre-treatment cortical thickness in the right inferior frontal gyrus. These findings provide initial insights into the structural neurobiology of FCD and its associations with symptom severity and clinical trajectories.

## Highlights

• Initial study of structural MRI alterations in functional cognitive disorder.
• Functional memory symptom severity correlated with right amygdala volume.
• Clinical improvement related to baseline right inferior frontal gyrus thickness.

## 1 Introduction

Functional cognitive disorder (FCD), a cognitive subtype of functional neurological disorder (FND), is characterized by distressing and potentially disabling cognitive symptoms that occur in the absence of macroscopic structural neuropathology (e.g., focal brain lesions) (Ball et al., 2020, Bhome et al., 2019, Cabreira et al., 2025a, McWhirter et al., 2020, McWhirter et al., 2022, Silverberg and Rush, 2024, Stone et al., 2015). The memory and other cognitive difficulties in FCD exhibit features of internal inconsistency, e.g., a mis-match between the severity of subjective complaints and objective cognitive performance (Ball et al., 2020). A recent systematic review revealed that approximately 24% of patients in memory clinics may have FCD (McWhirter et al., 2020). Head injury is among the most commonly reported acute precipitants, alongside post-viral sequelae and migraine (Cabreira et al., 2025b). Up to 50% of individuals who suffer a concussion experience persistent memory and other cognitive symptoms a year after injury (Machamer et al., 2022, Rioux et al., 2022, Theadom et al., 2016). Despite its prevalence and significant impact on daily functioning, the neural mechanisms underlying FCD are not well established.

The proposed neuropsychological mechanisms underlying other FND subtypes more broadly offer insights into the development and persistence of FCD, particularly in the context of head injury. Theoretical models of FND highlight the role of aberrant attentional processes, symptom-focused hypervigilance, and avoidance behaviors in the persistence of symptoms, which likely also contribute to FCD (Burke and Silverberg, 2025, Espay et al., 2018a, Teodoro et al., 2018). Predictive processing models suggest that acute injury may lead to the development of maladaptive predictions that are not properly updated via sensory inputs, thereby leading to symptom persistence (Edwards et al., 2012, Jungilligens et al., 2022a, Van den Bergh et al., 2017). Thus, in the case of FCD following concussion, cognitive symptoms initially attributable to head injury could be perpetuated and heightened through biased attentional and predictive mechanisms.

In the broader FND neuroimaging literature, these conceptual models are supported by the identification of heterogeneous alterations in brain regions across the default mode, salience/ventral attention, and somatomotor networks (Baizabal-Carvallo et al., 2019, Bègue et al., 2019, Drane et al., 2021, Perez et al., 2021, Westlin et al., 2025a), supporting the conceptualization of FND as a disorder of aberrant multi-network brain communication. Although functional differences are often highlighted as the predominant alterations in FND, recent evidence has also revealed subtle structural alterations across these networks (Bègue et al., 2019, Maurer et al., 2018, Westlin et al., 2025b). In particular, several studies have identified a relationship between grey matter alterations and symptom severity in those with functional motor disorder and functional seizures (Aybek et al., 2014a, Jungilligens et al., 2022b, Perez et al., 2018, Sojka et al., 2022). While the networks implicated across studies of other FND subtypes may also be relevant for FCD, no study to-date has investigated the structural neural correlates underlying FCD.

The present study aimed to address this gap by conducting a whole-brain structural magnetic resonance imaging (MRI) investigation of cortical thickness and subcortical volume in individuals with FCD after concussion compared to post-concussion controls. Including a post-concussion control group without FCD allowed us to account for the non-specific neurobiological effects of head injury, and to more specifically interrogate the structural correlates associated with FCD. In addition to between-group comparisons, we also investigated structural correlates of functional memory symptom severity and symptom improvement in individuals who underwent cognitive behavioral therapy (CBT) or cognitive rehabilitation. Based on prior findings in other FND populations and conceptual models of FCD (Burke and Silverberg, 2025, Cabreira et al., 2023b, McWhirter et al., 2020), we hypothesized that structural differences in FCD participants would be identified in default mode and/or salience/ventral attention network nodes, and that regional structural alterations would be associated with measures of symptom severity. In exploratory analyses, we also hypothesized that baseline structural profiles in brain areas implicated in cognitive control and other executive functions – as has been shown in other skills-based treatments (Clark and Beck, 2010, Cole et al., 2014) – would relate to prospectively collected clinical outcomes.

## 2 Methods

Thirty-seven participants with FCD following a concussion (27 female, 10 male; mean age [SD] = 39.1 ± 12.2 years; Table 1) were prospectively recruited from two concussion clinics in the Greater Vancouver Area and from a list of participants who had completed a previous concussion research study and consented to be contacted for future research (Silverberg et al., 2022). At case conference meetings, neuropsychiatrists and neuropsychologists determined by consensus if each participant met published diagnostic criteria for FCD. Specifically, a panel of three neuropsychiatrists, one neuropsychologist, and one neuropsychiatry fellow reviewed all cases, with 3–5 panel members present at each meeting. Consensus decisions were based on information from prior clinical interviews, brain imaging, and neuropsychological test results. Diagnostic criteria were as follows: (1) one or more cognitive symptoms, (2) evidence of internal inconsistency, (3) symptoms not better explained by another medical disorder, and (4) symptoms that cause clinically significant distress or impairment, or warrant medical evaluation (Ball et al., 2020, Cabreira et al., 2023a, McWhirter et al., 2020). Examples of internal inconsistencies are reported in Supplementary Material. Panel members judged cognitive difficulties to not be better explained by comorbid anxiety and/or depression when the onset, course, nature, and/or severity did not align with those conditions. Cognitive difficulties that did not temporally track with anxiety/depression, were characterized by internal inconsistencies, and exceeded what would be expected from these disorders alone were considered to support an FCD diagnosis. Differences in expert judgment were resolved through group discussions to achieve consensus. Eligibility criteria also required that participants: (1) be between 18 and 65 years old, (2) be fluent in English, (3) sustained a mild traumatic brain injury in the previous 6–24 months, as defined by the World Health Organization (WHO) Neurotrauma Task Force (Holm et al., 2005), (4) demonstrate adequate test-taking effort by passing both the 21-Item Test (telephone-administered version; Iverson, 1998, Kanser et al., 2021) and Test of Memory Malingering (Tombaugh, 1997), and (5) have regular access to an internet-connected device. Exclusion criteria included unstable/serious medical conditions, unstable/severe mental illness, active drug use disorder, use of medications known to cause memory impairment, and MRI contraindications. While the presence of other FND subtypes was not systematically evaluated for in this FCD cohort, no individuals endorsed another FND subtype as the predominant source of disability.

**Table 1:** Demographic and psychometric characteristics of patients with functional cognitive disorder (FCD) after concussion and post-concussion control participants.

	FCD (N = 37) Mean ± SD or N	Post-Concussion Controls (N = 25) Mean ± SD or N	FDR corrected *p-*value
**Age (years)**	39.1 ± 12.2	39.5 ± 13.2	1.0
**Sex**	F: 27; M: 10	F: 13; M: 12	0.2
**Days Since Concussion**	442.9 ± 157.8	418.6 ± 124.2	0.6
**Psychotropic Medication Use**	Yes: 12; No: 25	Yes: 7; No: 18	0.8
**Loss of Consciousness**	Yes: 16; No: 21	Yes: 15; No: 10	0.3
**Employed or Student**	Yes: 20; No: 17	Yes: 20; No: 5	0.1
**Self-Reported Premorbid Concussion History (1 or more)**	Yes: 21; No: 16	Yes: 8; No: 17	0.1
**Self-Reported Premorbid Anxiety and/or Depression Symptoms**	Yes: 22; No: 15	Yes: 6; No:19	0.02*
**TOPF**	106.8 ± 11.8	110.2 ± 9.8	0.3
**FMDI**	66.0 ± 9.5	47.2 ± 10.1	<0.001*
**MMQ-S**	25.2 ± 7.9	46.3 ± 12.7	<0.001*
**GAD-7**	8.7 ± 5.2	3.0 ± 4.4	<0.001*
**PHQ-9**	10.4 ± 4.8	3.6 ± 5.5	<0.001*

*P* values reflect statistical significance between the FCD cohort and the control group after False Discovery Rate (FDR) correction for multiple comparisons. Asterisks indicate corrected *p*-values < 0.05. Statistical comparisons of continuous variables were done using a Mann Whitney *U* test, while comparisons of discrete variables were done using a Chi-Squared test. 3 FCD participants and 1 control had incomplete data. For each variable, counts of missing data were as follows: FMDI: 3 FCD; MMQ-S: 1 control; PHQ-15: 1 control. F, Female, M, Male; TOPF, Test of Premorbid Functioning (adjusted score); FMDI, Functional Memory Disorder Inventory – Long Version; MMQ-S, Multifactorial Memory Questionnaire- Satisfaction subscale; GAD-7, Generalized Anxiety Disorder-7; PHQ-9, Patient Health Questionnaire-9.

Twenty-five post-concussion control participants (13 female, 12 male; mean age [SD] = 39.5 ± 13.2 years; Table 1) were also recruited from the same sources. All control participants had sustained a concussion within the past 6–24 months, but did not report persistent cognitive symptoms and did not meet criteria for FCD. Inclusion and exclusion criteria were the same as for the FCD group, with the added exclusion of a FCD diagnosis. Two additional controls were enrolled but excluded because their MRI scans were not completed. All subjects signed informed consent, and the University of British Columbia Clinical Research Ethics Board approved this study (REB #H22-00233).

### 2.1 Neuropsychiatric characterization

All participants in the FCD cohort completed a Mini International Neuropsychiatric Interview (MINI) V.7.0.2, a structured diagnostic interview based on the Diagnostic and Statistical Manual of Mental Disorders, Fifth Edition (DSM-5) (included modules tested for comorbid current generalized anxiety disorder, major depressive disorder, panic disorder, post-traumatic stress disorder, and substance use disorder). FCD symptoms were measured using the Functional Memory Disorder Inventory – Long Version (FMDI): a 22-item self-report scale measuring how frequently respondents experience memory lapses, including inattentive-type (e.g., ask others to repeat what they have just said) and blocking on overlearned information (e.g., recall the spelling of a word they know well), on a four-point Likert scale (Schmidtke & Metternich, 2009). General memory concern was assessed using the Multifactorial Memory Questionnaire- Satisfaction subscale (MMQ-S): an 18-item self-report scale measuring how satisfied (vs. concerned) a person felt regarding their memory over the past two weeks on a five-point Likert scale, with higher values indicating higher memory satisfaction (Troyer & Rich, 2002). Participants also completed the Patient Health Questionnaire (PHQ-9; Kroenke et al., 2001) and General Anxiety Disorder-7 scale (GAD-7; Spitzer et al., 2006), as well as neuropsychological testing, including the Test of Premorbid Functioning (TOPF) to estimate their premorbid intellectual functioning.

When a questionnaire was missing a single item, we prorated scores to calculate a total score. Proration was applied to a total of 6 scores for the MMQ-S, 8 scores for the FMDI, 4 scores for the GAD-7, and 1 score for the PHQ-9, across timepoints. Proration was not applied for participants who were missing two or more items on an outcome measure (3 FCD, 1 control).

### 2.2 Treatment study

A subset of participants with FCD (*n* = 24) from the cohort described above took part in a subsequent treatment study: Clinicaltrials.gov #NCT05581810 (Rioux et al., 2024). Briefly, participants were randomly assigned to receive either CBT (*n* = 11) or cognitive rehabilitation (*n* = 13). For the CBT intervention, components included identifying negative influences on attentional capacity, education on how attentional biases can distort one’s sense of normal forgetting, intentional exposure to memory lapses, instruction on cognitive-reappraisal strategies, and relaxation strategy training. For the cognitive rehabilitation intervention, components predominantly included instruction in the use of various memory aids. See Rioux et al., 2024 for additional details regarding the rationale for the clinical trial design, as well as session-by-session outline of the skills-based treatment interventions. Both interventions consisted of eleven 50-minute videoconference sessions over an average of 109.4 ± 23.6 days. Participants completed a battery of questionnaires, including the FMDI and MMQ-S, at both pre- and post-treatment. On average, 30.2 days ± 19.2 elapsed between their initial MRI scan and the start of treatment.

### 2.3 MRI acquisition and preprocessing

Imaging was performed for all subjects on the same Philips Ingenia Elition 3TX scanner using a 32-channel phased-array head coil. A high-resolution 3D T1-weighted magnetization prepared rapid gradient-echo sequence was used with the following parameters: flip angle = 8°, repetition time = 2400 ms; echo time = 4.3 ms, inversion time = 950 ms, slices = 225, field of view = 256 x 256 x 180 mm, resolution = 0.8 mm isotropic with compressed SENSE factor of 3.6 for a total acquisition time of 5:50. T1-weighed scans were preprocessed with *FreeSurfer* 7.3.2 software using the recon-all processing stream, as described previously (Dale et al., 1999). All scans were visually inspected to ensure good quality. Cortical reconstructions and subcortical segmentations were manually checked for accuracy and no scans required manual editing. For cortical thickness maps, a Gaussian kernel of 10 mm full-width half-maximum was applied. For subcortical data, volumes of 7 bilateral regions (putamen, caudate nucleus, globus pallidus, nucleus accumbens, thalamus, amygdala, hippocampus) were calculated using *FreeSurfer*’s automatic segmentation and normalized to total estimated intracranial volume from *FreeSurfer* at the individual subject level (Fischl et al., 2002). These regions are the primary subcortical grey matter structures segmented by *FreeSurfer.* Post-hoc analyses of amygdalar nuclei used volumes of 7 nuclei (lateral, basal, accessory basal, central, medial, cortical, paralaminar), also normalized to each subject’s total estimated intracranial volume (Saygin et al., 2017).

### 2.4 Cortical thickness and subcortical volumetric analyses

To assess differences in cortical thickness between patients with FCD and controls, we employed vertex-wise two-class general linear models (GLMs) in *FreeSurfer*. Statistical significance was determined using cluster-wise correction for multiple comparisons, applying a cluster-forming threshold of *p* < 0.001 and a cluster-wise threshold of *p* < 0.05. Differences in subcortical volumes between FCD patients and controls were evaluated using separate logistic regression analyses implemented in Python (v3.11.5). *P*-values for each regression were corrected for multiple comparisons using false discovery rate (FDR) correction (*p* < 0.05).

To evaluate the relationship between memory measures (i.e., FMDI, MMQ-S) and cortical thickness, we conducted one-class vertex-wise GLMs in *FreeSurfer*, with the same cluster-wise correction thresholds as above. Associations between memory measures and subcortical volumes were examined using separate linear regressions, with FDR correction applied. These analyses were performed within the FCD cohort alone, as well as trans-diagnostically across the FCD and the post-concussion control groups, to capture both distinct and shared relationships along a continuum of memory complaints.

To assess the relationship between treatment-related changes in memory measures (i.e., absolute change in FMDI, MMQ-S scores from pre- to post-treatment), we repeated the same cortical thickness and subcortical volume analyses with absolute change scores as covariates of interest. These analyses were restricted to the subset of FCD participants who received treatment, with treatment type and baseline FMDI or MMQ-S scores included as covariates in the primary models.

All analyses were adjusted for age, sex, loss of consciousness (yes/no), and estimated IQ. For statistically significant findings following multiple comparison corrections, post-hoc analyses additionally controlled for: (1) PHQ-9 (depression) scores; (2) GAD-7 (anxiety) scores; and (3) psychotropic medication use (yes/no).

## 3 Results

### 3.1 Demographic and psychometric comparisons

The FCD and post-concussion control cohorts did not differ on age, sex, days since concussion, loss of consciousness, employment, premorbid concussion history, estimated IQ, or psychotropic medication use (see Table 1**,** Supplementary Table 1). Compared to controls, patients with FCD scored higher on the FMDI, GAD-7, and PHQ-9, scored lower on the MMQ-S (reflective of lower memory satisfaction), and more FCD participants reported pre-injury anxiety and/or depression symptoms for which they were diagnosed and/or treated (59.5 % (n = 22) and 24.0 % (n = 6), respectively). Twenty-four participants with FCD met criteria for at least one concurrent psychiatric comorbidity according to the MINI.

### 3.2 Between-group structural MRI differences

There were no whole-brain corrected cortical thickness or FDR-corrected subcortical volumetric differences between the FCD and control cohorts.

### 3.3 Structural MRI associations with memory measures

**Fig. 1:**
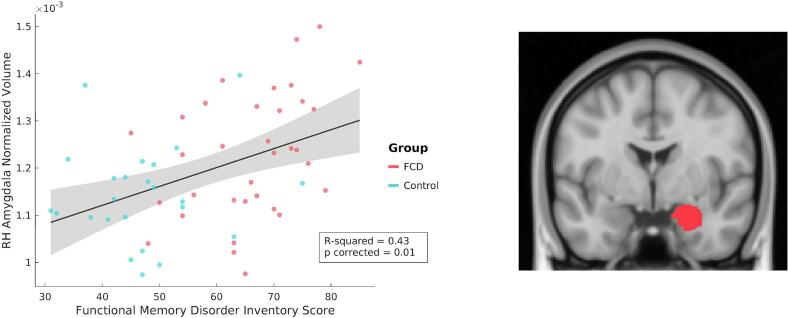
Functional memory symptoms, as measured by the Functional Memory Disorder Inventory (FMDI), positively correlated with the volume of the right amygdala (p_corrected_ = 0.01) across functional cognitive disorder (FCD) and post-concussion control cohorts. Outliers were removed prior to computing the correlation coefficients if they had a value exceeding more than 1.5 times the interquartile range above the upper quartile or below the lower quartile. (Left) For visual display purposes only, a scatter plot of normalized volumes (not adjusted for covariates of non-interest) is plotted against individual FMDI scores. Values for patients with FCD are shown in red, and post-concussion controls are shown in blue. (Right) Visualization of the right amygdala using the *FreeSurfer* aseg atlas. RH indicates right hemisphere. (For interpretation of the references to colour in this figure legend, the reader is referred to the web version of this article.)

**Fig. 2:**
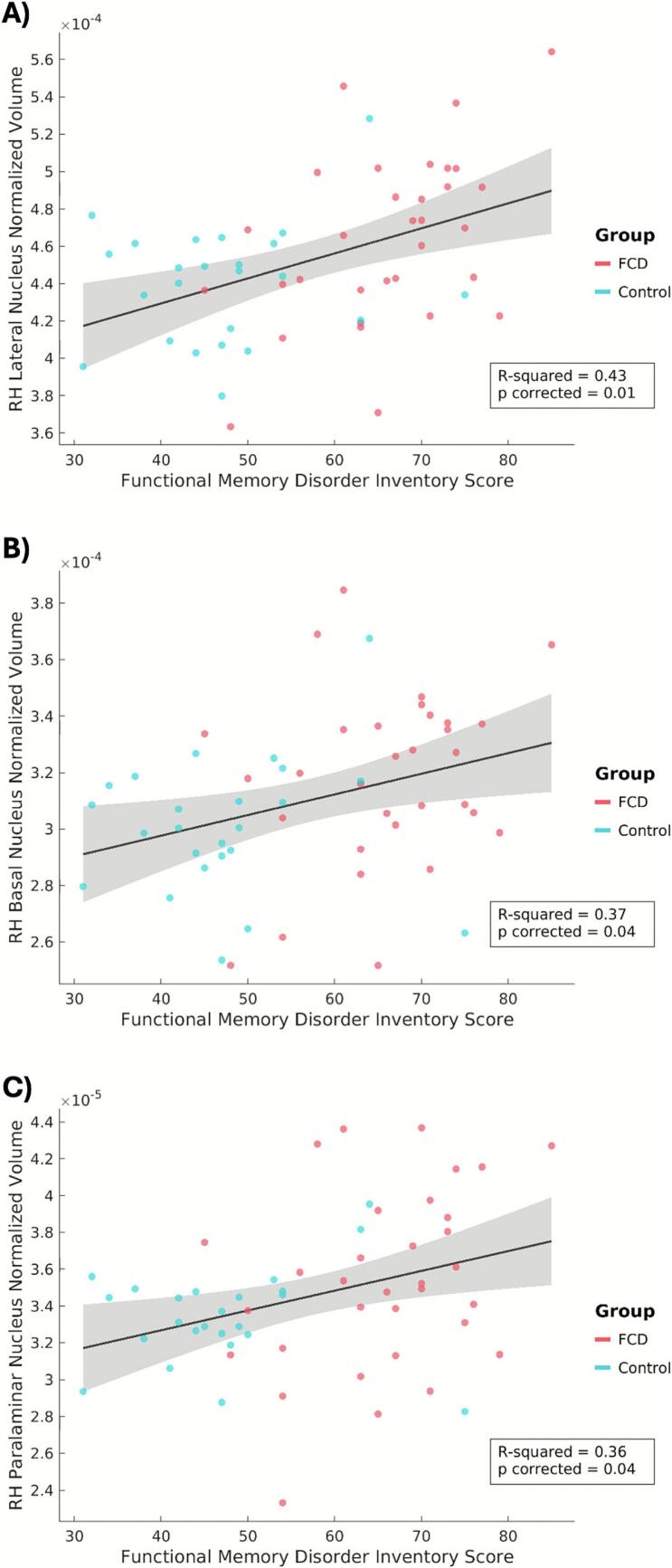
*Post-hoc* analyses revealed functional memory symptoms, as measured by the Functional Memory Disorder Inventory (FMDI), positively correlated with the volume of three amygdalar nuclei across functional cognitive disorder (FCD) and post-concussion control cohorts: (A) right lateral nucleus (p_corrected_ = 0.01), (B) right basal nucleus (p_corrected_ = 0.04), (C) right paralaminar nucleus (p_corrected_ = 0.04). Outliers were removed prior to computing the correlation coefficients if they had a value exceeding more than 1.5 times the interquartile range above the upper quartile or below the lower quartile. For visual display purposes only, scatter plots of normalized volumes (not adjusted for covariates of non-interest) are plotted against individual FMDI scores. Values for patients with FCD are shown in red, and post-concussion controls are shown in blue. RH indicates right hemisphere. (For interpretation of the references to colour in this figure legend, the reader is referred to the web version of this article.)

There were no significant associations identified within the FCD cohort alone. When looking trans-diagnostically across FCD and control participants, there was a significant positive association between individual FMDI scores and right amygdalar volumes (*p*_corrected_ = 0.008; Fig. 1). These findings held across all *post-hoc* adjustments for PHQ-9 scores (*p*_corrected_ = 0.02), GAD-7 scores (*p*_corrected_ = 0.003), and psychotropic medication use (*p*_corrected_ = 0.0003). *Post-hoc* analyses of right amygdalar nuclei revealed significant associations between FMDI scores and the right lateral (*p*_corrected_ = 0.01), basal (*p*_corrected_ = 0.04), and paralaminar (*p*_corrected_ = 0.04) nuclei (Fig. 2). Amygdalar nuclei findings held across all *post-hoc* adjustments, except when adjusting for PHQ-9 scores for the basal and paralaminar nuclei (Supplementary Table 2). There were no other significant associations between FMDI or MMQ-S scores across the FCD and control cohorts.

### 3.4 Exploratory predictors of treatment outcomes

**Fig. 3:**
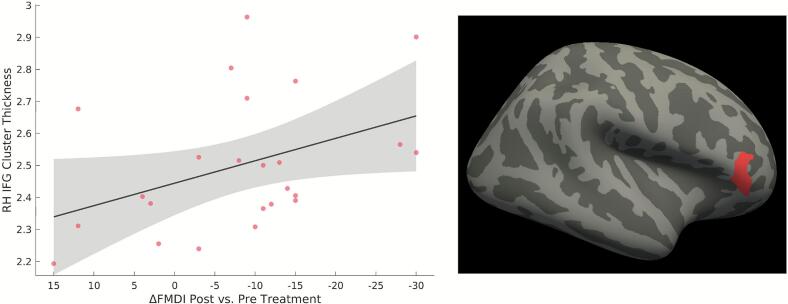
An improvement of functional memory symptoms following treatment (either cognitive behavioral therapy or cognitive rehabilitation), as measured by lower absolute change scores on the Functional Memory Disorder Inventory (FMDI), was associated with relative increases in pre-treatment cortical thickness of a right inferior frontal gyrus (IFG) region (p_corrected_ = 0.02) in patients with functional cognitive disorder (FCD); analyses were adjusted for type of treatment received. (Left) For visual display purposes, a scatter plot of cortical thickness volumes for the statistically significant right IFG region (not adjusted for covariates of non-interest) is plotted against individual FMDI scores. The x-axis values are reversed to reflect an improvement of symptoms from left to right. (Right) Visualization of the statistically significant right IFG cluster. RH indicates right hemisphere.

In participants with FCD who underwent treatment (either CBT or cognitive rehabilitation), there was a significant positive relationship between improvement in FMDI scores and greater right inferior frontal gyrus (IFG)/pars triangularis cortical thickness pre-treatment (*p*_corrected_ = 0.02, see Fig. 3). This finding held when adjusting *post-hoc* for PHQ-9 (*p*_corrected_ = 0.002) and GAD-7 (*p*_corrected_ = 0.047) scores, but did not hold when adjusting for pre-treatment psychotropic medication use. There were no significant associations with absolute change scores for the MMQ-S.

## 4 Discussion

This structural MRI study investigated cortical thickness and subcortical volumetric differences in individuals with FCD following a concussion. No significant structural differences were found between FCD and post-concussion control participants, suggesting that FCD is not characterized by gross neuroanatomical abnormalities, which aligns with the broader literature on other FND subtypes (Aybek et al., 2014a, Sojka et al., 2022, Zelinski et al., 2022). However, studies of FND have increasingly identified subtle structural differences in brain regions across the default mode, salience/ventral attention, and somatomotor networks – including when probing individual differences related to indices of symptom severity (Bègue et al., 2019, Perez et al., 2018, Sojka et al., 2022, Westlin et al., 2025b). Consistent with this, a significant positive association was observed between functional memory symptom severity (as measured by the FMDI) and right amygdalar volume across both the FCD and post-concussion controls. Post-hoc analyses revealed that this association was driven by individual differences in the volumes of the lateral, basal, and paralaminar nuclei of the amygdala. Additionally, in the subset of FCD participants who underwent treatment (CBT or cognitive rehabilitation), greater improvement in FCD symptoms was associated with relatively greater cortical thickness in the right IFG pre-treatment (controlling for the type of treatment received). These findings provide initial insights into the neurobiological processes underlying functional cognitive symptoms and highlight potential structural markers of symptom severity and treatment response.

The observed positive correlation between amygdalar volume and functional memory symptom severity – a finding that remained significant when controlling for depression scores, anxiety scores, and psychotropic medication use – suggests a potential link between this region, its associated connections, and the perceived memory difficulties encountered in patients endorsing functional cognitive symptoms. While the amygdala is not situated within a single resting state network, studies have demonstrated its connectivity with multiple large-scale networks, including the salience/ventral attention and default mode networks (Bickart et al., 2012, Bzdok et al., 2013, Elvira et al., 2022, Kerestes et al., 2017, Roy et al., 2009), consistent with our hypotheses regarding structural alterations within these networks. Though traditionally conceptualized as being involved in emotion processing, particularly in relation to threat perception and responses, the amygdala plays a broader role in detecting and directing attention towards salient sensory signals (Barrett, 2018, Blackford et al., 2010, Cunningham and Brosch, 2012; David Sander et al., 2003, Pessoa, 2010, Pourtois et al., 2013, Todd et al., 2012, Todd and Manaligod, 2018, Zheng et al., 2017). In other words, the amygdala helps to identify important changes in the external and internal (bodily) environment and alerts the rest of the brain to attend to and learn from these signals. A relationship between amygdalar volume and functional memory symptom severity may therefore reflect a heightened attentional bias and sensitivity to perceived changes in cognitive performance, leading individuals to more readily notice minor memory lapses and perceive them as threatening. Notably, this association was driven by differences in the lateral, basal, and paralaminar nuclei — amygdala nuclei that support a variety of functions, including the integration and weighting of affectively salient sensory signals, as well as the updating of future predictions based on these signals (deCampo and Fudge, 2012, McDonald, 2020, Polepalli et al., 2020). Given the observed associations between sensory nuclei of the amygdala (i.e., basolateral nuclei), future structural and functional connectivity research should investigate potential links between thalamo-amygdala connectivity, FCD, and persistent sensory symptoms post-concussion (McCombs et al., 2024). It also remains unclear whether the observed association with symptom severity is disease-related, a compensatory adaptation, or reflective of a pre-existing structural vulnerability that predisposes individuals to developing functional cognitive symptoms. In particular, because the observed relationship was present across both FCD and post-concussion controls, amygdala structure may vary with the degree of functional cognitive symptoms experienced rather than representing an FCD-specific feature. This pattern may reflect a continuum of symptom expression following head trauma, and further research is needed to distinguish mechanisms that specifically relate to FCD versus those that relate more broadly to persistent post-concussive symptoms.

The observed association between the amygdala and functional memory symptom severity in FCD is consistent with an extensive literature implicating amygdala structure and function across other FND subtypes. While findings have been in varying directions, differences in whole amygdala volume and its nuclei have been reported across several cohorts with functional motor disorder and/or functional seizures (Maurer et al., 2018, Nasrullah et al., 2023, Weber et al., 2023). Moreover, increased right amygdalar volumes were associated with worse mental health and trait anxiety symptoms in FND (Perez et al., 2017). Functional neuroimaging studies in FND have demonstrated heightened amygdala activity when viewing affective stimuli (Aybek et al., 2015, Hassa et al., 2017, Voon et al., 2010), as well as altered amygdalar functional connectivity, particularly with motor control areas (Aybek et al., 2014b, Diez et al., 2019, Espay et al., 2018b, Hassa et al., 2017). Of particular relevance, reduced habituation to affective stimuli in FND has also been reported (Aybek et al., 2015, Voon et al., 2010), potentially indicating heightened sensitivity to stimuli due to aberrant precision weighting of prediction errors. Our observed association between amygdalar volumes and severity of patient-perceived memory difficulties aligns with the perspective that FCD symptoms may arise from an increased biased attention towards minor cognitive fluctuations.

In exploratory analyses, we identified preliminary evidence that structural brain differences may be associated with treatment outcomes in FCD. Specifically, among participants who underwent a skills-based treatment (either CBT or cognitive rehabilitation), greater improvement in FMDI scores was associated with relative increased pre-treatment cortical thickness in the right IFG. This region falls within the broader frontoparietal network, which supports executive control – a relevant function for engaging with and benefiting from cognitive interventions (Cole et al., 2013, Dosenbach et al., 2006, Miller and Cohen, 2001, Stokes et al., 2013). Notably, imaging studies across several psychiatric and neurological disorders have implicated frontoparietal regions in psychotherapy outcomes (e.g., Clark and Beck, 2010, Cole et al., 2014, Kircher et al., 2013). For example, increased grey matter volume in the ventrolateral prefrontal cortex has been observed in individuals with chronic fatigue following CBT (de Lange et al., 2008), and was associated with decreased pain catastrophizing following CBT in those with chronic pain (Seminowicz et al., 2013). Similarly, right prefrontal cortical thickness has been linked to response to CBT in obsessive compulsive disorder (Bertolín et al., 2023). These findings highlight the role of frontoparietal regions, and specifically the IFG, in cognitive processes that are likely important for therapeutic success. Beyond its traditionally conceptualized role in executive control, the frontoparietal network is theorized to be involved in modulating predictions about incoming sensory signals (Barrett, 2017). In the context of FCD, greater cortical thickness in regions of this network may therefore reflect an enhanced capacity to engage in the process of updating maladaptive predictions or re-interpreting normal memory fluctuations in a less threatening manner. Thus, our preliminary evidence suggests that individual differences in cortical thickness within frontoparietal regions may contribute to treatment responsiveness in FCD, although further validation is needed to assess its predictive utility at the individual level. Given that most patients in this exploratory cohort improved following either receipt of CBT or cognitive rehabilitation, future research should also include a larger and more heterogeneous sample of patients with FCD to further investigate structural MRI predictors of clinical outcomes.

This study has several limitations. The sample size was modest, particularly for treatment analyses, which prevented a specific focus on treatment type. Future studies with larger samples and longitudinal data collection are needed to better understand the relationship between brain structure, symptom severity, and treatment response. Such work will help clarify whether the observed associations reflect stable traits, predisposing vulnerabilities, or experience-dependent neuroplastic changes. In the absence of a non-treatment control group, we also cannot determine whether observed associations reflect a treatment-specific effect or a marker of individual differences in the natural course of symptom evolution; future research is needed to provide additional clarification. The present study focused on adults who developed FCD following concussion. There are other precipitants of FCD, such as viral infections and migraine (Cabreira, ert al., 2025b). As such, more research is needed to discern whether the observed relationships are found in patients with FCD precipitated by non-head trauma factors. We had limited information about participants’ health and life events, and the absence of an age- and sex-matched healthy control group limits our ability to contextualize structural findings relative to healthy normative values. Furthermore, resting-state functional connectivity data was not collected, and thus additional research is needed to investigate functional connectivity changes in FCD.

In conclusion, this is the first MRI study investigating structural brain alterations and associations with clinical improvement in FCD. We observed a relationship between individual differences in FCD symptom severity and right amygdalar volume, with specific involvement of the lateral, basal, and paralaminar nuclei. We also identified an association between improvements in FCD symptoms following skills-based treatment and baseline cortical thickness differences in the right IFG. These findings set an initial foundation for future efforts aimed at comprehensively delineating the neural networks implicated mechanistically in FCD and its treatment responsiveness.

## CRediT authorship contribution statement

**Christiana Westlin:** Writing – review & editing, Writing – original draft, Visualization, Methodology, Formal analysis, Conceptualization. **Mathilde Rioux:** Writing – review & editing, Investigation, Data curation. **Jaqueline Lee:** Writing – review & editing, Investigation, Data curation. **William Panenka:** Writing – review & editing, Data curation. **Daniela J. Palombo:** Writing – review & editing. **Rebecca M. Todd:** Writing – review & editing. **Noah D. Silverberg:** Writing – review & editing, Supervision, Project administration, Funding acquisition, Data curation, Conceptualization. **David L. Perez:** Writing – review & editing, Supervision, Data curation, Conceptualization.

## Funding

This project was primarily funded by the 10.13039/501100021813WorkSafe BC Specific Priorities Research Grant (#RS2021-IG09) as awarded to N.D.S.; C.W. and D.L.P. were supported by R01MH125802 (10.13039/100000025NIMH).

## Declaration of competing interest

The authors declare the following financial interests/personal relationships which may be considered as potential competing interests: D.L.P. has received honoraria for continuing medical education lectures; royalties from Springer for a functional movement disorder textbook and honoraria from Elsevier for a functional neurological disorder textbook; is on the editorial boards of *The Journal of Neuropsychiatry and Clinical Neurosciences* (paid), *Brain and Behavior* (paid), *Epilepsy & Behavior*, and *Cognitive and Behavioral Neurology*; has received funding from the Sidney R. Baer Jr. Foundation and the Warren Alpert Foundation unrelated to this work; and is on the FND Society Board of Directors and American Neuropsychiatric Association Advisory Council.

## Data Availability

For qualified researchers, analysis code and de-identified data pertaining to study results can be made available upon reasonable request and following approval by the local internal review board.
