# Supplementary material for: Structural brain alterations and predictors of clinical improvement in functional cognitive disorder after concussion
Source: Neuroimage Clin. 2025 Dec 19;49:103936. doi: 10.1016/j.nicl.2025.103936 (PMC12811585; doi:10.1016/j.nicl.2025.103936)
Supplement: Supplementary Data 1 [file mmc1.docx]

**Supplementary Materials**

**Supplementary Methods**

Diagnostic criteria for FCD includes evidence of internal inconsistency. Examples of internal inconsistencies (as detailed in Ball et al., 2020, McWhirter et al., 2020) included:

1. subjectively-reported cognitive difficulties and/or low standardized cognitive test scores that markedly contrasted with (a) conversational abilities, (b) reported activities, (c) collateral accounts of cognitive functioning, or (d) other aspects of the clinical presentation;
2. inconsistencies between reported symptoms and performance on neuropsychological tests;
3. patterns on testing suggesting that certain cognitive processes performed better when accessed less explicitly (1 standard deviation).

**Supplementary Table 1. Self-reported employment status across patients with functional cognitive disorder (FCD) after concussion and control samples.**

|  | **FCD** (N = 37)  **N (%)** | **Controls** (N = 25)  **N (%)** |
| --- | --- | --- |
| **Not working or going to school for reasons unrelated to injury** | 5* (13.5%) | 1 (4.0%) |
| **Not working or going to school because of injury** | 2* (5.4%) | 0 (0%) |
| **Working or going to school, but in a less demanding way (e.g., reduced hours or classes) compared to before injury** | 11 (29.7%) | 4 (16.0%) |
| **Working or going to school, just as prior to injury** | 20 (54.1%) | 20 (80.0%) |

*One participant selected both ‘not working’ items, suggesting they are not working for various reasons.

**Supplementary Table 2. Statistical significance of primary and post-hoc analyses relating memory measures to right amygdalar nuclei across all study participants.**

|  | **Primary analysis**  *p*_corrected_ | **+ PHQ-9**  *p*_corrected_ | **+ GAD-7**  *p*_corrected_ | **+ Psychotropic Meds**  *p*_corrected_ |
| --- | --- | --- | --- | --- |
| **Lateral** | 0.010 | 0.008 | 0.004 | 0.001 |
| **Basal** | 0.039 | 0.068 | 0.043 | 0.003 |
| **Paralaminar** | 0.039 | 0.201 | 0.043 | 0.010 |

*P* values reflect statistical significance of the association between Functional Memory Disorder Inventory – Long Version (FMDI) scores and normalized amygdalar nuclei volumes after False Discovery Rate (FDR) correction for multiple comparisons across all 7 nuclei. Primary analyses adjusted for age, sex, loss of consciousness, and estimated IQ. Post-hoc analyses also adjusted for Patient Health Questionnaire-9 (PHQ-9) scores, Generalized Anxiety Disorder-7 (GAD-7) scores, and psychotropic medication use (yes/no).
